# From roots to glamor: gendered transformations of adapted folk songs in contemporary Bollywood

**DOI:** 10.3389/fsoc.2026.1747205

**Published:** 2026-05-29

**Authors:** Marziya Begum, Akaitab Mukherjee

**Affiliations:** Division of English, School of Social Sciences and Languages, Vellore Institute of Technology, Chennai, India

**Keywords:** adaptations, Bollywood, commodification, folk songs, gender, hybridity, male gaze, objectification

## Abstract

**Introduction:**

The research investigates how 21st century Bollywood adaptations of regional folk songs transform gendered meanings through narrative reorientation, visual spectacle and cultural hybridization. It examines how folk songs often rooted in women's voices and communal memory are reframed within commercial cinematic logic, reshaping the ways femininity, desire and modernity are represented on screen.

**Methodology:**

A qualitative, multimodal thematic analysis was conducted on four prominent Bollywood adaptations, namely, “Jugni” (2011), “Engine Ki Seeti” (2014), “Pallo Latke” (2017) and “Genda Phool” (2020). Each music sequence was treated as a multimodal text (lyrics, camera, mise-en-scène, choreography, and costume). Close viewings, lyric transcriptions and time-stamped visual annotations were used to generate codes and refine three core themes.

**Findings:**

The analysis shows that Bollywood's adaptations of folk songs undergo major gendered transformations. Traditional, community-based meanings are recast into individualized romance, spectacle and commercial appeal. Female objectification remains central, as camera work, choreography, costume and lyrics consistently sexualize women even when presenting them as confident or modern. Cultural hybridity, seen in “Hinglish” lyrics, westernized styling and the preference for fair-skinned performers, further commodifies folk authenticity for urban and global audiences. In the case studies, “Jugni” becomes a celebration of a modern woman narrated through male admiration; “Engine Ki Seeti” sexualizes an introspective folk narration; “Pallo Latke” shifts from playful bridal folklore to consumerized sensuality; and “Genda Phool” removes a socially rooted narrative and converts it into glossy pop glamor.

**Discussion:**

Folk to Bollywood adaptations operate as ideological acts that preserve and circulate regional songs while simultaneously distorting their cultural meanings to serve patriarchal and market logics. While occasional moments of staged agency appear, they are often subsumed within visual regimes that prioritize consumption and male pleasure. From a sociological standpoint, the paper advocates for more ethical adaptation practices, including transparent crediting and community collaboration. It calls for future empirical studies on audience interpretations, production cultures and intersectional dynamics. Such work can deepen understanding of how popular media reconfigures cultural memory, gender relations and regional identities in contemporary India.

## 1 Introduction

“… ‘Bollywood,' is one of the best-known and most widely appreciated features of contemporary Indian culture. It makes its presence felt beyond the screen throughout wider media, whether the Internet, television, news, art, literature, or music…” (Dwyer, 2014).

Bollywood, described as “a free-floating signifier” (Gera and Chua, 2012), has generated considerable scholarly discourse, with debates centering on its definition, scope and function. The term gained prominence in the post-liberalization era of the 1990s and early 2000s, coinciding with India's economic globalization and the increasing transnational circulation of Hindi cinema. Prior to this period, the umbrella term “Hindi cinema” was more commonly used to describe the mainstream film industry based in Mumbai. Over time, however, “Bollywood” has evolved into a multifaceted concept. It is understood as an industry operating from Mumbai and as a cultural conglomerate encompassing films, television, magazines, websites, fashion, music, and dance (Ganti, 2012; Rajadhyaksha, 2003). Globalization has facilitated the circulation of cultural products across national borders, enabling them to reach transnational audiences. Within this context, Bollywood has emerged as the primary representative of Indian cinema on the global stage. Bollywood's unique blend of melodramatic structures, centrality of love and elaborate song-and-dance sequences has allowed it to travel seamlessly across linguistic and cultural boundaries, finding audiences far beyond South Asia (Krämer, 2016). Viewers from diverse parts of the world, across Africa, the Middle East, Europe and North America, regularly engage with Bollywood through cinema halls, streaming platforms or social media. This transnational popularity not only showcases India's soft power but also reveals how Bollywood serves as a medium for cultural exchange and identity formation among diasporic communities.

Unlike Hollywood musicals, where songs generally serve to advance the storyline, Bollywood songs frequently function as spectacular displays of rhythm, choreography, costumes, and emotion, rather than following narrative logic (Morcom, 2007). These sequences extend beyond the films themselves, becoming cultural artifacts. Even when a film underperforms commercially, its songs often achieve independent success, circulating widely through radio, television and digital platforms. Since the early 2000s, numerous instances illustrate the growing success of Bollywood music in the international market. One of the most visible indicators of this global reach is YouTube viewership. Indian music labels such as T-Series and Zee Music Company dominate digital streaming platforms, consistently ranking among the most subscribed and viewed channels worldwide. For example, in June 2025 the Zee Music channel exceeded 100 million subscribers, making T-Series and Zee Music Company among the very few music companies to achieve this milestone on YouTube (Patil, 2025). Another dimension of this global success is observed on music streaming platforms like Spotify, where Indian artists have seen significant growth in international listenership. The Indian Express (2025) reports that playback singer Arijit Singh achieved 134.2 million followers outranking many international pop stars. Moreover, according to Spotify's own report (Spotify Newsroom, 2025), streams of Indian artists in international markets surged by over 2,000% between 2019 and 2023, and in 2024 nearly half of the royalties earned by Indian artists on Spotify came from listeners outside India. Global recognition of Bollywood songs has also led to a rise in international collaborations. Artists such as A.R. Rahman have worked with international musicians like Andrew Lloyd Webber, The Pussycat Dolls and Mick Jagger. Priyanka Chopra's collaborations with American artists like Pitbull demonstrate the crossover potential of Indian music talent. Similarly, the global remix culture has seen international DJs and producers incorporating Bollywood sounds and samples into mainstream pop and electronic music. These collaborations highlight how Bollywood continues to influence global pop music aesthetics.

To adjust to the evolving demands of global entertainment markets, remix and adaptation are recurring practices in Bollywood. Older songs are frequently revived with new arrangements. For instance, songs such as “Laila Main Laila” (2017) and “Aankh Marey” (2018) are contemporary reworkings of classics from the 1980s and 1990s. These revivals draw on nostalgia to attract multigenerational audiences. Because the original songs are copyrighted, Bollywood producers typically secure rights from the music labels or composers before reusing them. However, such remix culture also reflects the industry's reliance on recognizable sounds and collective memory to ensure commercial success. This paper, however, does not focus on remix culture but rather on the adaptation of folk songs into 21st century Bollywood. Adaptations are not mere copies but rather creative, interpretive acts of appropriation and re-contextualization (Hutcheon, 2006). Historically, folk songs have served as vital carriers of regional traditions, oral histories and collective cultural memory (Montagu et al., 1969). By adapting these songs, Bollywood transforms them with modern orchestration, glamorous visuals and star-centered choreography (Booth and Shope, 2014). For instance, the Rajasthani folk song “Kesariya Balam” has appeared in several films, including *Dor* (2006), where it retains its cultural poignancy, and *Padmaavat* (2018), where it is stylized to fit a grand historical spectacle. “Navrai Majhi,” Maharashtrian folk songs, appears in *English Vinglish* (2012) with vibrant wedding choreography and festive orchestration. These adaptations illustrate how Bollywood borrows from folk culture to evoke regional authenticity while simultaneously commodifying it for cinematic spectacle. Unlike old film songs, folk compositions are often community creations that lack identifiable ownership or written documentation. As a result, Bollywood filmmakers seldom require copyright permissions when incorporating them. Bollywood plays an important role in preserving folk traditions by introducing them to younger, globalized audiences who may be disconnected from their cultural heritage. For example, “Bumbro” (2000) brought Kashmiri folk song to mainstream cinema viewers at a time when the region was largely represented through narratives of political conflict (Ganti, 2012). Folk music in India has been transmitted orally over generations, evolving through regional performances and local storytelling traditions. When these songs are adapted in Bollywood, they are frequently detached from their original cultural significance and reinterpreted for mass entertainment. By turning folk songs into polished cinematic spectacles, their local significance is often diminished, reducing them to consumable products for urban and international audiences (Srinivas, 2002). This tension between cultural preservation and commercialization forms a central concern of this paper. Moreover, these adaptations frequently reshape the gender dynamics embedded within folk songs. In many cases, women are subjected to objectification through altered lyrics and the emphasis on visual spectacle, even when the folk songs in their original contexts carried communal or celebratory meanings. Such reworkings introduce new negotiations of representation and identity.

Gender operates as a central analytical lens in understanding the transformation of folk songs within contemporary Bollywood. While many regional folk traditions historically articulated women's voices, desires and collective experiences within community contexts, their cinematic adaptations frequently reconfigure these meanings through commercial aesthetics and visual spectacle. In the process, the female body is often repositioned as a site of display, shaped by performance conventions that privilege visibility, desirability and consumption. Drawing on feminist film theory, particularly the notion of the male gaze, these adaptations can be read as producing gendered subjectivities that align with patriarchal and market-driven ideologies. Thus, the paper takes a critical stand that the movement of folk songs from community spaces to mainstream cinema not only alters their cultural context but also reshapes their gendered significance, raising critical questions about representation, agency and the politics of cultural translation in a globalized media environment.

## 2 Review of literature

The following review maps two strands of literature in Bollywood: studies of song circulation and the adaptation of folk materials, and feminist film scholarship on objectification and the male gaze. Bringing these literatures together clarifies why folk to Bollywood adaptations are not merely musical choices but ideological acts with specific gendered transformations.

### 2.1 Songs, folk traditions, and adaptation in Bollywood

Beyond entertainment, songs define how Bollywood articulates identity and emotions. Song sequence has remained at the core of Bollywood aesthetic and continuously adapt to the changing tastes of audiences (Gehlawat and Dudrah, 2017). Bollywood songs often detach from film narratives to circulate as independent cultural commodities consumed in both domestic and diasporic contexts (Morcom, 2007; Gopal and Moorti, 2008). A distinct feature of Bollywood's musical evolution is its deep engagement with folk traditions. Bollywood thrives on fusion drawing from classical, folk and popular songs to craft hybrid soundscapes (Booth and Shope, 2014). Bhabha (1994) posits that hybridity is a “third space” of negotiation where colonial and indigenous elements merge, creating new, ambivalent identities. Bollywood's hybrid soundtracks, mixing Punjabi dhols with EDM beats or inserting English phrases into Hindi lyrics, reflect such negotiations between local authenticity and global modernity (Pudaruth, 2015). Folk melodies and lyrics serve as foundational materials for cinematic reinvention, allowing Bollywood to merge regional authenticity with commercial modernity. Folk songs are transmitted across generations as performative expressions of regional belonging and social commentary (Agarwal, 2012). These songs often emerge from collective experiences of labor, love, celebration and resistance, particularly within agrarian and women's traditions. Scholars have highlighted how particular filmmakers use folk music as narrative texture and political commentary. Bajwa and Singh (2022) demonstrate that Vishal Bhardwaj's use of folk songs in his films exemplifies how folk elements can deepen regional identity and emotional realism. His work shows that Bollywood can preserve and modernize folk songs meaningfully. Yet, much of the scholarship on Bollywood's use of folk songs remains preoccupied with issues of fusion, authenticity and preservation, without examining how these adaptations transform the gender politics embedded in the originals. Bollywood acts as a major stage for transmitting India's musical diversity to the world. However, this circulation often comes at the cost of erasing the socio-cultural contexts of folk songs, especially those centering women's voices. When folk or devotional music becomes part of commercial media, it is transformed into a visually oriented form of entertainment, shifting the focus from sound to spectacle (Srinivas, 2002). Hutcheon (2006) emphasizes that adaptation is not imitation but a creative and interpretive process involving recontextualization and cultural negotiation. Similarly, Sanders (2006) argues that adaptations often reveal the ideological anxieties of their time, as they mediate between the “source” and the “target” culture. In Bollywood, these adaptations tend to transform local, context-rich folk expressions into pan-Indian or global commodities, aligned with the industry's commercial and aesthetic imperatives (Ganti, 2012; Booth and Shope, 2014). This duality, of preservation and appropriation, mirrors what Storey (2018) terms the commodification of culture, wherein cultural expressions lose their original social meaning as they are repackaged as consumable entertainment. The adaptation of folk songs in Bollywood thus reconfigures them from community-oriented expressions to commodified performances shaped by market logics and patriarchal gazes.

### 2.2 Objectification and male gaze in Bollywood

**Figure 1:**
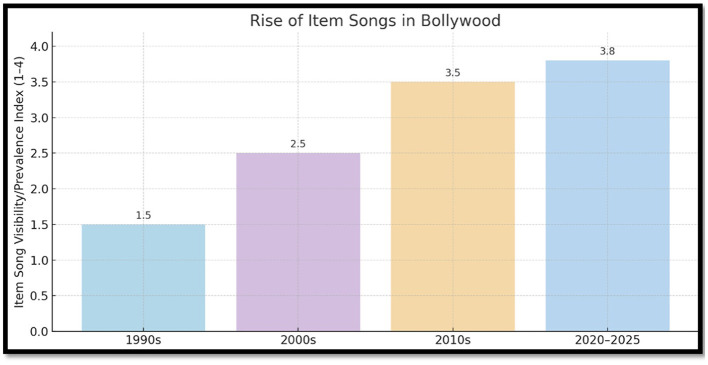
Literature-based trend index developed from secondary sources about rise of item songs in Bollywood.

Parallelly, a substantial body of feminist film scholarship has critiqued the objectification of women in Bollywood. The chart (Figure 1) highlights the rising prominence of item songs from the 1990s to 2025, showing their shift from minor additions to major commercial features of Bollywood (The Juggernaut, 2022; The Federal, 2024). Women's increased screen presence in commercial cinema did not translate into agency, rather, they are re-inscribed within patriarchal stereotypes of beauty, devotion and sacrifice (Butalia, 1984). Subsequent studies have extended this critique to song sequences, where women's bodies are foregrounded as visual attractions (Gokulsing and Dissanayake, 2004; Mehta, 2017). Mulvey's (1975) concept of the male gaze, explains how classical film structure positions the woman as a spectacle to be looked at and the man as the bearer of the look. This visual pleasure is sustained by cinematic techniques such as camera framing, slow motion and fragmentation of the female body, all of which reinforce patriarchal pleasure structures. Later scholars have applied Mulvey's framework to analyze Bollywood's item numbers and music videos, arguing that these sequences function as sites of male fantasy rather than female empowerment (Mitra, 2013; Dwyer, 2014). Further analyzing item songs, arguing that women are commodified both lyrically and visually through metaphors, innuendo and bodily display, reinforcing heteronormative desire (Ain et al., 2019; Vaidya, 2021). Fredrickson and Roberts's (1997) objectification theory complement Mulvey's analysis by linking visual representation to psychological consequences. They argue that constant objectification leads women to internalize an outsider's perspective on their bodies, resulting in self-surveillance and reduced agency. Contemporary feminist media studies link the persistence of this objectification to intertwined structures of patriarchy, capitalism, and technology. Choudhury and Sharma (2025) argue that male-dominated creative control, digital platforms and audience complicity perpetuate sexist imagery in Bollywood. Similarly, Imam (2024) finds that Bollywood item songs reinforce patriarchal ideologies, depicting women as vain and submissive while glorifying male dominance. From a socio-legal standpoint, such representations not only perpetuate inequality but also violate constitutional rights to dignity and equality, contributing to normalized gender violence (Chaudhary and Nanda, 2025). Together, these frameworks reveal how Bollywood songs reinforce patriarchal hierarchies through songs.

### 2.3 Research gap

Existing scholarship on Bollywood music and gender has largely developed along two parallel trajectories: studies of item songs and studies of folk adaptation. While both bodies of work offer important insights, they remain insufficiently integrated, leaving a significant gap in understanding how gender operates within folk song adaptations.

A substantial body of research has examined the representation of women in Bollywood item songs, particularly through the lens of objectification and the male gaze. Studies by Kamble and Biswal (2022), Purohit (2019), and Vaidya (2021) demonstrate that item numbers function as sites of sexual objectification, where women's bodies are fragmented, eroticized and commodified for visual pleasure. These works consistently argue that item songs reinforce patriarchal ideologies and normalize gender inequality through spectacle-driven cinematic techniques. Similarly, Shewade (2023) highlight how item songs contribute to the construction of oppressive gender roles and influence societal perceptions of femininity by presenting women as objects of desire within male-dominated visual frameworks. However, this strand of scholarship is largely confined to item numbers as a distinct genre, often detached from narrative context and treated as isolated spectacles. As a result, it does not sufficiently account for how similar mechanisms of objectification operate within other musical forms in Bollywood, particularly those that originate from culturally embedded traditions such as folk songs.

Parallel to this, another body of literature has explored Bollywood's engagement with folk music in terms of hybridity, authenticity and cultural circulation. Scholars such as Booth and Shope (2014), Ganti (2012), and Morcom (2007) have examined how Bollywood appropriates and transforms regional musical traditions to cater to global and diasporic audiences. Drawing on frameworks of cultural hybridity (Bhabha, 1994) and adaptation theory (Hutcheon, 2006), these studies emphasize processes of fusion, commodification and transnational circulation. While they provide valuable insights into the aesthetics and politics of adaptation, they largely overlook how these transformations reshape gendered meanings embedded within folk traditions, especially those historically rooted in women's voices and lived experiences.

Moreover, existing studies tend to adopt either textual analysis (lyrics) or visual analysis (spectacle) in isolation. There is limited research that systematically integrates multiple modes, lyrics, camera work, choreography, costume and performance to examine how gender is constructed across cinematic layers. This lack of a multimodal analytical framework restricts a comprehensive understanding of how meaning is produced in Bollywood song sequences.

Therefore, a critical gap emerges at the intersection of gender studies, adaptation studies and multimodal analysis. Specifically:

- Studies on item songs identify objectification, but do not extend this analysis to folk adaptations.
- Studies on folk adaptations analyze hybridity and commodification, but neglect gender transformation.
- Existing research rarely examines how multiple cinematic elements interact simultaneously to produce gendered meaning.

This paper addresses these gaps by bringing together feminist film theory and adaptation studies within a multimodal analytical framework. By analyzing four contemporary Bollywood adaptations of folk songs, it demonstrates how cultural recontextualization is inseparable from gendered transformation, revealing that folk adaptations are not neutral cultural transfers but ideological sites where femininity, desire and modernity are actively reconstructed.

## 3 Methods

This study employed a qualitative research design grounded in thematic analysis to explore how four 21st century Bollywood adaptations of folk songs rework original narratives and produce gendered representations, particularly female objectification, through lyrics, visual framing, choreography and costume. The analytic procedure followed Braun and Clarke's (2006, 2019) guidance for reflexive thematic analysis, treating each music video as a multimodal text in which verbal (lyrics) and visual (camera, mise-en-scène, costume, color, and choreography) modes interact to produce meaning. The study was theoretically informed by Laura Mulvey's concept of the male gaze (1975) and objectification theory (Fredrickson and Roberts, 1997). Complementary concepts of cultural hybridity and adaptation (Bhabha, 1994; Hutcheon, 2006) were used to interpret linguistic mixing, westernizing tendencies and processes of commodification observed in the adaptations. The goal was not statistical generalization but a systematic, close reading that links micro-level formal choices to broader discourses of gender, modernity and cultural appropriation (Bordwell and Thompson, 2016).

### 3.1 Materials

The selection of these four songs was guided by a purposive and theoretically informed sampling strategy. Bollywood has adapted a wide range of folk songs across regions, including popular examples such as “Nimbooda Nimbooda” (*Hum Dil De Chuke Sanam*, 1999), “Bumbro” (*Mission Kashmir*, 2000), “Navrai Majhi” (*English Vinglish*, 2012), and “Morni Banke” (*Badhaai Ho*, 2018). While these songs are significant in demonstrating the industry's engagement with folk traditions, many of them function primarily as festive, celebratory or performance-oriented sequences that do not centrally articulate women's subjectivity or lived experience. In contrast, the four songs selected in this study originate from folk traditions that foreground women's voices, emotions, and social realities. This distinction is crucial to the study's analytical focus, as it enables a more precise examination of how female-centered narratives are transformed when recontextualized within Bollywood's visual and commercial frameworks. Additionally, the selected songs are temporally distributed across the 21st century, enabling the study to capture evolving patterns in Bollywood's adaptation practices over time rather than reflecting a single moment or trend. Together, these songs span different regional traditions (Punjabi, Rajasthani, and Bengali), production contexts (film-based and independent music video) and representational strategies, enabling a comparative analysis of gendered transformation. Rather than aiming for statistical generalization, the study prioritizes analytical depth and conceptual variation, selecting cases that most clearly illustrate the broader patterns under investigation. This approach aligns with qualitative research traditions that emphasize information-rich cases to illuminate complex cultural processes (Patton, 2002). The four adapted songs, each representing distinct strategies of adaptation, were:

- “Jugni” from *Tanu Weds Manu* (2011); full video song (approximately 3 min). Source: YouTube (official upload).
- “Engine Ki Seeti” from *Khoobsurat* (2014); full video song (approximately 4 min). Source: film file (viewed from downloaded copy).
- “Pallo Latke” from *Shaadi Mein Zaroor Aana* (2017); full video song (approximately 4 min). Source: YouTube (official upload).
- “Genda Phool” by Badshah feat. Payal Dev (2020); music video (approximately 3 min). Source: YouTube (official upload).

The secondary material for this study comprises a range of textual and critical sources that supports the primary analysis. These included the original folk songs and their published lyric transcriptions where available, as well as news articles that addressed issues of authorship, provenance and controversy surrounding these specific adaptations. In addition, scholarly works on film form, song culture, objectification, hybridity, and adaptation were consulted to provide theoretical and contextual grounding for the analysis. All video materials were accessed through official streaming platforms or authorized uploads and the corresponding lyric sources were cross verified to ensure transcriptional and translational accuracy.

### 3.2 Procedure

The analytic procedure followed a structured, multi-stage process and established cinematic close-reading techniques (Braun and Clarke, 2006; Bordwell and Thompson, 2016). Each song sequence was viewed multiple times to develop contextual familiarity. The study uses purposive sampling, emphasizing in-depth close readings of four selected songs to privilege interpretive depth and the findings were therefore framed as interpretive rather than generalizable. Initial viewings helped establish the narrative and tone, while subsequent viewings focused on identifying visual elements related to gender representation and adaptation. The researchers, proficient in Hindi and Bengali, were able to engage directly with the lyrical and cultural nuances of most of the selected songs. For other regional folk songs, assistance was sought from native speakers and supplemented by online translation tools to ensure an accurate understanding of the folk lyrics. The lyrics of both the Bollywood adaptations and their folk sources were transcribed and cross-checked against subtitle files and lyric sources, with English translations produced for analytical comparison when necessary. Salient visual moments were captured through screenshots and annotated with time stamps to facilitate multimodal analysis. Verbal and visual data were then segmented into meaningful analytical units, such as lyric lines that reframed folk tropes, camera angles that fragmented the female body and costume or choreography choices that suggested hybridity. This process was conducted reflexively, recording both observable features and underlying symbolic meanings (Braun and Clarke, 2019). The resulting codes were organized into themes and refined in alignment with the theoretical framework. Three key thematic categories emerged: narrative shift (folk meanings reoriented toward romance and spectacle), female objectification and the male gaze (fragmentation, camera focus, and lyrical voice) and white-skin obsession and westernization (linguistic mixing, hybridity, and commodified modernity; Mulvey, 1975; Fredrickson and Roberts, 1997; Bhabha, 1994). The final phase involved a multimodal close reading in which lyrical analysis was integrated with visual interpretation of camera framing, mise-en-scène, choreography, and costume to illustrate how different modes work together to produce gendered meanings (Bordwell and Thompson, 2016). All empirical observations were interpreted through Mulvey's (1975) concept of the male gaze, Fredrickson and Roberts's (1997) objectification theory, Hutcheon's (2006) theorie of adaptation, and Bhabha's (1994) of hybridity, situating the cinematic techniques within broader sociocultural discourses.

## 4 Finding and analysis

All four Bollywood adapted folk songs examined in this study have achieved significant popularity among audiences. “Jugni” stood out primarily because it creatively reinterpreted a familiar Punjabi folk refrain (Roy, 2011). Although “Engine Ki Seeti” received a mixed critical response, it established itself as a promotional highlight for the film, attracting attention through its engaging visuals and folk fusion appeal (Adivarekar, 2014). The success of “Pallo Latke” appears to stem from its social resonance, as it became a “wedding anthem” and a lively dance number inspired by traditional folk elements (Javaid, 2023). Meanwhile, “Genda Phool” gained immense popularity not only for its wide audience reach but also due to the controversy surrounding the lack of credit given to its original composer, Ratan Kahar (Jain, 2020). This controversy further boosted the song's visibility through widespread media and public discussion. After analyzing the songs, the recurring themes identified are discussed in the following sub-sections.

**Figure 2:**
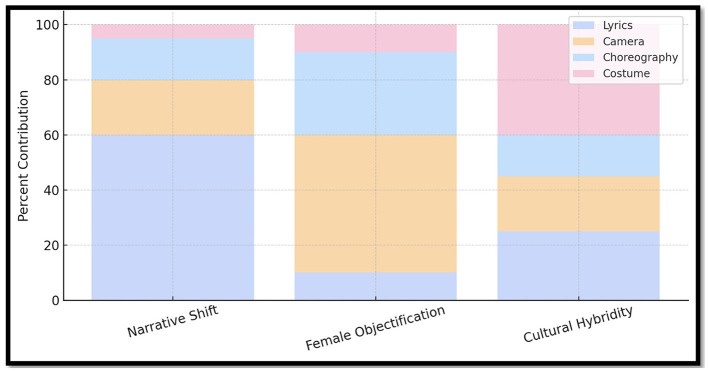
Multimodal contribution to thematic construction in adapted folk songs.

To illustrate how different cinematic elements collectively shape thematic meaning in the adapted folk song sequences, a multimodal contribution chart was created by coding four key modalities, namely, lyrics, camera work, choreography, and costume, across all selected adapted folk songs and estimating their relative influence on the three dominant themes identified in the study (Figure 2). Each modality was assessed through close viewings, time-stamped annotations and qualitative intensity coding. The coded percentage values were then organized and plotted using standard data-visualization tool to present each theme as a composite of interacting modalities. The resulting chart demonstrates that narrative shift is primarily driven by lyrical recontextualization, female objectification relies heavily on camera techniques and choreography aligned with the male gaze and cultural hybridity emerges from the interplay of Hinglish lyrics, fusion choreography and westernized costuming shaped by globalized aesthetics. By presenting themes as multimodal rather than purely lyrical, the figure underscores how Bollywood's adaptation of folk songs operates through layered aesthetic choices that collectively reproduce patriarchal, commercial and hybridized meanings.

### 4.1 Narrative shift

Folk songs, across cultures, often serve as repositories of collective memory, narrating the cultural, historical, and social practices of a community. They function as a medium to preserve and transmit the traditions of folk people through oral performance, allowing the continuity of cultural identity across generations. The narratives embedded in folk songs frequently reflect the daily lives of the people. For instance, in many communities, songs are sung during labor-intensive tasks such as fishing, shepherding, farming, or other forms of manual work (Alam et al., 2023). These work songs not only help synchronize group efforts but also provide a rhythmic focus, easing the burden of repetitive labor (Nettl, 2005). Another significant narrative strand in folk songs revolves around historical stories, celebrating the courage, valor and sacrifices of great heroes and martyrs. Such songs often serve both as a form of remembrance and as a moral guide, teaching values such as bravery, loyalty, and social responsibility. Nature frequently emerges as a symbolic element in folk songs, reflecting human emotions and experiences (Pande, 2019). Rivers, mountains, forests, and seasons are not just backdrops but also metaphors for love, longing, grief or joy. Among these themes, love is particularly pervasive (Oza, 2020); folk songs explore the nuances of romantic emotions, personal admiration and the joys or pains of relationships, often embedding these experiences within the natural landscape. In the 21st century, folk songs are increasingly adapted into films and albums, but the lyrical narratives often undergo a shift to resonate with globalized audiences. This adaptation goes beyond musical arrangement as it alters the very stories these folk songs once carried. Through these adaptations, the essence of folk songs as carriers of oral history and social identity is diluted (Patil, 2025). The lyrical focus shifts from community-based storytelling, rituals or satire to individual romance, fun or spectacle, aligning with the demands of a globalized entertainment industry.

#### 4.1.1 Shift in representation

“Jugni” is a famous folk song sung at Punjabi weddings. The word “Jugni” has a strong historical background in India. It carries layered meanings across regions and timelines (Kochhar, 2020). It is a Punjabi folk term that literally translates to female firefly. Sometimes the word is also used to describe an ornament or piece of jewelry worn by women. However, its symbolic meaning goes far beyond that. In traditional Punjabi folk songs, “Jugni” is not merely an insect, she becomes a metaphor for the soul, spirit or consciousness that moves freely through the world, witnessing and commenting on human life, joy, suffering and social realities. During the Sufi era, though there is no concreate proof of the folklore, in some narrative strands, “Jugni” symbolized the pain of separation, a young girl traveling from one village to another in search of her lover but unable to reunite with him, embodying both longing and spiritual wandering. Most who believe this love-story version of “Jugni” vaguely claim that it might have originated anywhere between the mountains of Kashmir in the north and the sands of the Cholistan Desert (now in Pakistan) in the south. Then followed by this revolutionary “Jugni” appears to have its roots in the Revolt of 1857. It was sung in praise of Indian sepoys. The most credible theory relating to the origin of “Jugni” dates to 1908 to two Punjabi folk singers Manda and Bishna (Rana, 2013). During the 50th anniversary of Queen Victoria's reign, the Jubilee flame was taken to every district headquarter of every state under the British Empire. In Punjab, the revolting sentiments were brewing, but still at a nascent stage. The poets, Bishna and Manja followed the procession of the Jubilee flame and sang songs channeling the anger of the people against the establishment. “Jugni” might be the corrupt form of the English word “jubilee.” So impressive was their music that many people would come out in abound to see them perform. Their growing popularity landed the poets in trouble, and they were arrested by the British police. However, the most popular and celebrated version of “Jugni” dates to the 1960s by the famous Pakistani-Punjabi singer Alam Lohar (Asia Society, 2012) who, for the first time, recorded “Jugni” in electronic form. He used the word “Jugni” to highlight the one's understanding of the world and relationship with God. Few lines from Alam Lohar's “Jugni” (Kochhar, 2020) went like this:

“Ae way Allah waliyan di Jugni Ji,

Ae way nabi pak di Jugni Ji,

Ae way Maula Ali wali Jugni Ji,

Ae way mere peer di Jugni Ji,

Ae way saaray saba di Jugni Ji.”

(Translation: “Jugni is the spirit of God,

Jugni is the spirit of the Holy Prophet,

Jugni is the spirit of Ali and his followers,

Jugni is the spirit of my saints,

She is the spirit of all His words.”)

Following these many variations of “Jugni” like cultural expression, social critique and spirituality, contemporary Bollywood provides a different dimension to this folklore. It has been adapted, in Bollywood many times, in films such as *Oye Lucky! Lucky Oye!* (2008), *Tanu Weds Manu* (2011), *Queen* (2013), and *Highway* (2014) where the term is recontextualized as a marker of rustic charm, sensuality, or free-spirited femininity (Baig, 2021).

For discussion in this paper, the adaptation of the folk song “Jugni” appearing in the climax of the film *Tanu Weds Manu* is examined. It is solely dedicated to honor the female protagonist of the film. Tanuja Trivedi (Kangana Ranaut), known simply as Tanu, is one of the most dynamic and unconventional female characters in contemporary Bollywood. She embodies a blend of rebellion, freedom and emotional complexity, standing out sharply against the traditional portrayals of idealized heroines that dominated earlier Hindi films (Singh and Kumari, 2024). From her first appearance, Tanu is presented as a free-spirited and impulsive young woman from Kanpur who refuses to be confined by conservative social norms. She drinks, smokes, parties and openly challenges patriarchal expectations that defy the typical image of the Indian bride. When her parents arrange her marriage to the gentle and reserved doctor, Manu (R. Madhavan), she rebels immediately, declaring that she already has a boyfriend. This act of defiance sets the tone for her characterization as someone who values autonomy and emotional honesty, even when it leads to chaos. However, her impulsiveness often masks vulnerability and fear of emotional dependence. While she mocks Manu's simplicity, she is simultaneously drawn to his sincerity. Her decisions are driven more by emotion than logic and she oscillates between independence and the desire for love. This emotional volatility makes her a realistic, flawed and human character, rather than a romantic ideal. She symbolizes the modern Indian woman in transition, caught between traditional expectations and the urge for individuality (Kumar, 2023). Her personality mirrors the shifting sensibilities of urban middle-class India, where women increasingly seek self-definition beyond marriage and respectability. In feminist readings, Tanu represents a woman learning to negotiate freedom within patriarchal constraints, often stumbling but refusing to submit.

This adapted folk song sung by Mika Singh serves as a cinematic tribute to the character of Tanu, reflecting a deliberate transformation of the traditional “Jugni” motif to represent the modern Indian woman. In its original form, the folk song often conveyed themes of emotional pain or devotion, usually voiced by a spirit expressing their innermost desires. However, in its Bollywood adaptation, the narrative shift significantly, celebrating freedom, individuality and feminine strength. In the adapted version, “Jugni” is reimagined as a spirited, bold and carefree woman who embodies both tradition and modernity. She is depicted as someone unafraid to challenge norms, speak her mind and assert her independence (T-Series, 2011; 00:00:20–00:00:35). Her vivacious personality sometimes seen as mischievous is symbolic of the way patriarchal societies often label outspoken women. Yet, beneath this surface lies a deeper portrayal, a woman who is educated, self-reliant and unafraid to fight for what she believes in. This duality challenges conventional patriarchal expectations, presenting her not as a rebel without cause, but as a complex individual driven by conviction and authenticity. The song further humanizes this “modern woman” by emphasizing her emotional depth and capacity for love. Despite her independence, she still seeks genuine emotional connection and true love, a quality traditionally associated with idealized women in Indian society. Through this, the song bridges the gap between the “woman with agency” and the “woman of virtue,” showing that empowerment and emotional sincerity can coexist. This evolution mirrors the experiences of contemporary Indian women who navigate the contradictions of modern relationships where dating apps and fleeting “situationships” coexist with a deep yearning for meaningful companionship. Bollywood's adaptation, therefore, not only redefines “Jugni” but also celebrates the evolving identity of Indian women. It positions such women as worthy of admiration rather than judgment, emphasizing that strength, wit and independence do not negate traditional values. The song's closing reference to *Jugni* being in London extends this representation beyond national borders, suggesting that the essence of the modern Indian woman resonates even within the diaspora (T-Series, 2011, 00:02:50–00:03:05). This globalized framing allows the Indian diaspora to embrace and celebrate a contemporary, empowered version of “Jugni,” blending nostalgia with progress. Ultimately, the song honors both the folk roots of “Jugni” and the transformed identity of the modern Indian woman, reflecting how Bollywood uses adaptation not merely as entertainment but as a tool for reinterpreting gender, identity and tradition in the context of a changing world. In contrast to “Jugni,” the next two adapted folk songs reveal how Bollywood tends to sexualize and impose gendered interpretations on traditional songs, resulting in a significant transformation of their original narratives.

#### 4.1.2 Recontextualizing female experience

The Marwari folk song “Anjan Ki Seeti” originates as a lyrical narration sung by a woman during her train journey. It captures the woman's sensory experiences and inner reflections as she observes the passing world. She speaks of her heart racing as the train moves quickly, and she pleads with the driver to slow down so she can savor the beauty around her. The song becomes a poetic meditation on travel, memory, and the fleeting sense of freedom it offers. Through her observations like the running animals, the whirring fan inside the train and a carefree girl walking with confidence, the singer reflects on her own limited agency as a woman (Khan, 2007). The contrast between the freedom of the animals and the girl's fearless stride underscores her realization that she once possessed greater freedom as a child than she does now as an adult woman constrained by social expectations. Her narration thus becomes a subtle expression of longing, nostalgia and self-awareness. Folk songs such as these often served as oral histories of everyday life for rural Rajasthani women, giving voice to their emotions, desires, and reflections on the world around them. The train here is symbolic of both progress and possibility, representing women's entry into public spaces and mobility during a time when travel itself was a privilege. The woman's song, therefore, becomes a metaphor for momentary liberation. She experiences freedom through motion even if it remains temporary.

In *Khoobsurat*, this folk song undergoes a radical transformation in the adapted version titled “Engine Ki Seeti.” The Bollywood adaptation shifts the meaning of the folk song from introspection and mobility to overt sexual innuendo. The original's meditative tone is replaced by a playful, energetic, and sensual performance. The film introduces this song early in the narrative, shortly after the protagonist, Milli Chakravarty (Sonam Kapoor), arrives at the royal palace as a physiotherapist. At this point in the story, the film contrasts Milli's bright, spontaneous nature with the formality and rigidity of the royal household. The palace represents silence, tradition and restraint, whereas Milli embodies freedom, laughter and emotional openness. “Engine Ki Seeti” marks the first disruption of that rigid world. Her uninhibited dancing and singing become a spectacle that shocks the conservative environment but also signifies the beginning of transformation within it. However, in the cinematic version, the lyrics and imagery of the folk song are heavily sexualized. The original woman's plea for the train to go slow which was a metaphor for savoring life's experiences is replaced with a command to go faster. The movement of the train, the sound of the whistle, and the rhythmic beats all take on erotic undertones. The lines:

“Phak phak engine bol raha hai,

Patri tharr tharr kaanpe,

Kahan rukegi gaadi aakar,

Kahan rukegi gaadi aakar mann yeh mera pooche…

Engine jud jaaye mujhse aake khaun hichkole…”

(Translation:

“The engine is making a loud noise,

There's a strong vibration on the track,

Where will this train stop,

Where will this train stop, my heart is asking…

As the engine connects with me, I feel the bump…”)

explicitly transform the train into a metaphor for sexual intercourse (Kashyap, 2014; 00:35:00–00:35:14). The repetitive, mechanical sounds of the engine and the trembling of the tracks evoke the sensations of physical passion. The phrase “engine jud jaaye mujhse” (Translation: “the engine connects with me”) intensifies the sexual imagery, suggesting a merging of bodies through rhythmic motion. The engine itself operates as a phallic symbol, representing male energy, desire and penetration, while the train track becomes the site of feminine receptivity.

What was once a woman's subjective reflection on freedom, in the original folk song, becomes a hypersexualized performance. Yet, within the film's broader context, the song also functions as an expression of Milli's liberated femininity. “Engine Ki Seeti” can be read as an assertion of her bodily confidence and agency. She dances openly, unapologetically owning her sexuality which is a sharp contrast to the silent, restrained royal family that surrounds her. Milli, as a modern Indian woman, refuses to suppress her emotions or desires to fit patriarchal ideals. Her dance becomes a moment of reclaiming space and celebrating freedom of expression. Through her, the film suggests that women's agency over their bodies and desires need not be hidden or shameful. However, this empowerment is complicated by the way the song is constructed. While Milli's actions embody confidence, the song's visual and lyrical framing still caters to a heteronormative male fantasy. This reflects Foucault's (1978) argument that modern societies constantly produce and reproduce discourses of sexuality not to liberate it, but to regulate and define it through coded language, imagery, and performance. The adapted folk song, therefore, exemplifies how popular cinema in Bollywood commodifies female sexuality under the guise of empowerment.

#### 4.1.3 Celebration to commodification

Another Marwari folk song, “Pallo Latke,” which celebrates femininity, undergoes a significant shift in meaning when adapted into Bollywood. Traditionally, this folk song is sung during festive occasions such as weddings and community gatherings, reflecting the spirit of joy, playfulness and collective celebration. The title “Pallo Latke” literally translates to “the swinging loose end of the veil.” “Pallo,” also known as “ghunghat,” holds deep symbolic, cultural and emotional resonance in Rajasthani society. It is not merely a piece of clothing but an emblem of a woman's identity, modesty, and social standing. The “Pallo” represents both beauty and the cultural constraints associated with traditional womanhood. In Rajasthani customs, the “Pallo” is the loose end of the veil that a woman drapes over her head, often using it to cover her face before elders or men as a gesture of dignity, grace, and respect (Mishra, 2017). The colors, embroidery, and style of draping signify various aspects of a woman's life like her age, marital status and community identity. Folk songs and dances from Rajasthan often use the swinging “Pallo” as a symbol of youthful vitality, charm and romantic expression, celebrating femininity within the boundaries of modesty and cultural propriety. The traditional lyrics of the song, “Mhara pallo latke re, mhara pallo latke/Dekho sajan ji, and mhara pallo latke” (Translation: “My veil's end is swinging, oh look beloved, my veil is swinging!”) capture the essence of teasing and innocent flirtation (Choudhury, 2014). The song is when performed as part of wedding celebrations, marks the transition of a young girl into married life. The playful exchange between a bride and her husband reflects affection and tenderness rather than overt sensuality. It symbolizes the beginning of womanhood and the emotional maturity associated with marriage.

When the song is adapted into Bollywood, the contextual framework largely remains the same, but the interpretation and presentation shift considerably. The adapted version appears after the climax scene in *Shaadi Mein Zaroor Aana*, where the story revolves around Aarti (Kriti Kharbanda) and Satyendra (Rajkummar Rao), a couple who fall in love through an arranged marriage. On the night of their wedding, Aarti runs away to pursue her career ambitions, leaving Satyendra heartbroken. Years later, fate reunites them as government officers. Satyendra is now bitter and seeks revenge, but eventually, they reconcile and decide to marry. The song “Pallo Latke” is used to celebrate their reunion and the wedding. However, while the surface context, marriage and celebration, remains unchanged, the underlying meanings are notably altered. In the Bollywood version, the opening lines immediately signal this shift. The woman's request for jewelry, a “20 g ring and 30 g bracelet,” places her within a transactional framework (Zee Music Company, 2017; 00:00:28–00:00:37). These lyrics objectify her within patriarchal and consumerist structures. The exchange of ornaments becomes emblematic of how women are commodified within the institution of marriage in India, reduced to decorative possessions to be adorned and exchanged. This reworking detaches the folk song's innocent playfulness and replaces it with a patriarchal trope in which the bride's worth is defined through material gifts and aesthetic display (Soni, 2020). The Bollywood song, therefore, transforms a communal folk expression of femininity into a spectacle centered on wealth and consumption. The woman in the adapted Bollywood song also displays a new kind of assertiveness. She takes pride in her appearance and even directs the romantic interaction, but this agency remains confined within a patriarchal frame that equates her confidence with sexual availability. Here, when she sings “Pallo Latke,” the veil is redefined as a marker of sexual readiness. The swinging “Pallo” here metaphorically announces her willingness to engage in intimacy, signaling her transition from emotional to physical union with her partner. This transformation of meaning marks Bollywood's tendency to reinterpret traditional symbols of womanhood through a lens of sensuality and spectacle. This shift becomes especially apparent in the song's final stanza:

“Raat bhar main paas tere badhne de craze re,

Dhadkano ko haule haule hone de tezz re,

Phal sabar ka meetha chocolate se zyada,

Kuch der tu rakh le naik iraada,

Maan ja na, na kar manmaani,

Boli jaave meri jawani,

Mere jalwo ka asar hai sabse hatke…

Pallo latke re mahro pallo latke,

Zara sa, zara sa, zara sa tedho hoja balma.”

(Translation:

“I am close to you all night, so let the craze grow,

Slowly allow your heartbeats to become fast,

The fruit of patience is sweeter than chocolate,

Hold on to your good intentions for some time,

Agree with me and don't be stubborn,

My youth continuously says that,

the magic of my charm is different from everyone…

My veil is dangling,

Become a little bit crazy, my beloved.”)

This final stanza clearly refers to the anticipation of sexual union (Tangri et al., 2017; 00:03:46–00:04:26). The imagery of the night, rising heartbeat and restrained desire evokes the sensual atmosphere of a couple about to engage in intimacy. The woman appears to lead the interaction as she asks her partner to be patient, to allow passion to build and to surrender to her youth and beauty. Superficially, this may seem to depict female sexual agency. However, within the Bollywood framework, this so-called empowerment often functions as a stylized fantasy constructed for male consumption. The woman's confident sensuality is choreographed, lit and performed in a way that reaffirms her role as an object of desire rather than a subject of self-expression. Thus, this stanza does not merely describe a sexual act, but it also reflects how Bollywood treats women in its visual and lyrical narratives. Female sexuality is presented as bold and liberated, yet it remains confined within patriarchal control. The woman's body and desire are not celebrated for her autonomy but are commodified as entertainment (Mitra, 2013). What once symbolized the graceful movement of a young bride's veil in celebration of life and love is transformed into a metaphor of erotic display and possession.

#### 4.1.4 Silencing the marginalized

Not only during celebrations, but we also have folk songs that narrate sad and sorrowful events. Folk songs have traditionally also been a way for communities to express shared emotions of grief and suffering, capturing experiences of loss, hardship, migration, separation, or social injustice (Chauhan and Mishra, 2024). Before the advent of modern media, music served as one of the few ways for people to preserve and communicate emotional experiences collectively. One such folk song that addresses social injustice and marginalization is the deeply rooted Bengali oral song “Boro Loker Biti Lo,” composed by Ratan Kahar (Ghosh, 2020). Beneath its simple, lyrical beauty lies a profound story of pain, betrayal and maternal love. The song narrates the life of a prostitute who falls in love with a rich man, only to be abandoned when she becomes pregnant. Their relationship, though filled with emotion, remains socially unacceptable and the woman is left to face the harsh realities of life alone. She gives birth to a daughter, and the song becomes her emotional address to her child. In the most evocative lines, “Bodo loker biti lo lomba lomba chul/Emon matha bendhe dibo lal genda phool” (Translation: “Daughter of a rich family with long hair/I will tie red marigold flowers in your hair”), the mother expresses her wish to decorate her daughter's hair with red marigolds (Kahar, 2020; 00:00:35–00:00:48), a gesture symbolic of her maternal pride and tenderness. The red marigold represents love, devotion and auspiciousness, but it also carries undertones of pain and sacrifice. The flower thus becomes both a ritual of love and an act of resistance. It is the mother's way of celebrating her daughter's existence despite the stigma attached to her birth. Alongside this expression of maternal love, the song also recalls memories of her time with her lover. The mention of red dust, characteristic of Bengal's landscape, becomes a poetic witness to her past and anchors her personal suffering within a specific regional and social context. The song ends with the mother's fear for her daughter's future, wondering what will happen to her child after her death. In this way, the folk song pays tribute to the lives of prostitutes, who are often victims of exploitation and neglect. Bengal, particularly Kolkata, continues to be one of India's major centers of prostitution (Mishra, 2016) and the song reflects the ongoing social injustice faced by marginalized women. Through its poignant narrative, the folk song becomes both a voice of empathy and a critique of societal hypocrisy, exposing the intersection of gender, class, and morality in Indian society.

When adapted in Bollywood, however, these emotional and moral layers are completely stripped away. The song “Genda Phool,” featuring Badshah and Jacqueline Fernandez, removes the pain and social commentary of the original and replaces it with a glamor and urban aesthetic. The Bengali folk lines are retained but woven into a composition filled with rap verses, heavy beats and glossy visual imagery. The music video visually evokes the atmosphere of Durga Puja, one of Bengal's grandest festivals. Its set design, costumes and dance sequences are full of Bengali cultural and festive elements. At the beginning of the video, a few “Chhau” dancers appear, followed by Badshah entering a vibrant celebration scene. The female lead, Jacqueline, along with a group of women dancers dressed in “Korial” (Laal Paar Sada) sarees and adorned with traditional jewelry, performs the “Dhunuchi” dance, a ritual performance linked to Durga worship. The video presents a sexualized portrayal of women, where camera movements, dance choreography and costume choices emphasize physical beauty. Badshah's rap verses are lustful and objectifying, as he openly admires the woman's body and movement through suggestive language.

“Bum tera gotay khaye,

Kamar pe teri butterfly,

Body teri makhan jaise…

Sanwla sa rang tera,

Bawla sa dhang tera,

Mujhse chori chori baatein kare ang ang tera…”

(Translation:

“Your bum is moving left and right,

There's a butterfly on your waist,

Your body is like butter…

Your color is dusky,

Your styles are amazing,

Every body part of yours talks with me secretly…”)

These lyrics make it evident that the Bollywood adaptation is centered on admiring the woman's physical beauty and expressing lustful attraction toward her (Badshah, 2020; 00:00:28–00:01:25). The woman's body becomes the site of desire, celebrated for its sensual appeal (Badshah, 2020; 00:00:25–00:00:34). Here, the woman appears to feel happy being admired and takes pride in her beauty, saying she will adorn herself with a red marigold flower. Yet, ironically, in the entire music video, Jacqueline's long hair is never adorned with a red marigold. The traditional imagery of tying red marigold flowers, which in the folk song symbolized a mother's love and resilience, is reinterpreted as a visual motif of sensuality and self-adornment. The transformation reveals how Bollywood often commodifies female representation, reducing complex narratives of suffering into spectacles of beauty and desire. The adaptation sparked controversy when it was revealed that Ratan Kahar had not been credited for the original folk composition (Mukherjee, 2020). The deeper issue extends beyond plagiarism, it reflects how cultural works from marginalized voices are appropriated and stripped of context in mainstream media (Storey, 2018). In this case, the folk song's social critique of gender and class oppression is replaced by a glamorous portrayal of female desirability, turning cultural history into a marketable aesthetic.

The pattern of narrative shift becomes evident in these four Bollywood adapted folk songs. While folk songs originally emerged from lived experiences, expressing women's emotions, struggles, and reflections on social realities, their Bollywood adaptations often alter these meanings to fit the demands of entertainment and mass appeal. In this process, the female voice that once articulated longing, freedom or pain is frequently replaced by objectification of women's bodies. Yet, these adaptations also offer a mirror to contemporary society, showing how gender, class, and cultural identity are negotiated in modern India.

### 4.2 Female objectification and male gaze

Female objectification refers to the process by which women are treated primarily as objects of visual or sexual pleasure rather than as autonomous agents with thoughts, desires or subjectivity. Objectification theory, developed by Fredrickson and Roberts (1997), explains that women are socialized to internalize an observer's perspective on their own bodies, leading to self-surveillance, body dissatisfaction and diminished agency. In media and cinema, objectification manifests when female characters are fragmented into body parts, sexualized in movement or costume or reduced to their aesthetic and erotic appeal rather than their role in the narrative. Objectification is closely tied to theory of the male gaze (Mulvey, 1975), which positions women as passive spectacles for male viewers while men remain active subjects who drive the story. Mulvey argued that mainstream cinema is structured around patriarchal values, positioning women as objects of visual pleasure while men are the bearers of the gaze. According to her, cinematic representation operates on three levels of gaze: the gaze of the camera, the gaze of the characters within the film and the gaze of the spectator. Each of these gazes is coded in ways that reinforce male subjectivity. The female form is stylized, commodified and eroticized for male pleasure.

In Bollywood, the male gaze has been prominent since the early decades of cinema. In the “golden age” of Hindi cinema (1950s−1970s), even as films addressed themes of social justice and national identity, female characters were framed through song-and-dance sequences where their beauty, sensuality or submissiveness was foregrounded. Songs like “Babuji Dheere Chalna” (1954) and “Roop Tera Mastana” (1969) used close-up shots of the heroine's face, lips and body movements to construct female desire for male viewers while rarely offering the woman an independent narrative voice. The rise of the so-called “item number” in the 1990s intensified female objectification in Bollywood. Songs such as “Choli Ke Peeche Kya Hai” (1993) and “Sheila Ki Jawani” (2010) epitomize Mulvey's framework by reducing women to fragmented body parts and innuendo. Here, the female figure is often divorced from the main plot and introduced solely to add spectacle, with lyrics that objectify women in overtly sexualized terms. These performances reinforce the commodification of the female body, turning women into entertainment products for the male gaze (Choudhury and Sharma, 2025). Over time, however, the tendency to sexualize female bodies became more explicit, especially as cinema became a mass-consumption medium competing for global markets. Contemporary examples such as “Fevicol Se” (2012) and “Kamariya” (2018) illustrate how the woman's body is central to the spectacle.

#### 4.2.1 Camera's gaze and the centrality of the female face

This dynamic continues to persist even in Bollywood's adaptations of folk songs. Female objectification and male gaze are evident in the visual representation of these songs. The camera in cinema constructs meaning and directs viewer's attention. Through camera positioning, movement and framing, directors decide what the viewer sees, how they see it and what they feel about it (Bordwell and Thompson, 2016). In all these four Bollywood folk adaptations, the camera frequently lingers on different parts of the female body, fragmenting and objectifying it for the pleasure of the viewer. There is a commonality in all these songs, there is always a scene where when a woman dances or sways her body, a slow motion and focus shot is provided (Zee Music Company, 2017, 00:00:55–00:01:02), which is never done for the male when he dances. Every time the woman is introduced there is emphasis on her face. She is recognized by her outer physical appearance. In “Jugni”, the faded camera lights are used to reveal different parts of her face (T-Series, 2011, 00:00:06–00:00:10) Similarly, in the song “Pallo Latke,” the woman's face becomes the central object of desire, and its beauty is foregrounded through both lyrical and visual elements. The lyrics emphasize how people are captivated merely by looking at the woman's face, reinforcing the cultural notion that a woman's worth and attractiveness are tied primarily to her physical appearance. The emphasis on external beauty echoes the traditional Hindu ritual of “Muh Dikhayi,” in which the bride's face is revealed. In this ritual, the bride's face is usually veiled, and the act of unveiling symbolizes both the revelation and evaluation of her beauty. This cultural reference is directly mirrored in the song, where the female character invites her beloved to lift her veil and witness the beauty of her face, further reinforcing the idea that unveiling is an act of display meant to draw male attention and admiration. It is accompanied by the lines: “Chehra mera piece Noorani/Dekhega toh kha jayega sau sau jhatka” (Translation: “My face is beautiful/You'll go crazy if you see it”; Zee Music Company, 2017, 00:00:52–00:00:58). The woman here is proud of her physical beauty and herself reduced her agency. Moreover, the lyrics attribute intoxicating power to the woman's face, as expressed in the line: “Thoda close to aaja baby, mukhda dikha ja/Bina peete hi mujhe to yaaron chadhne lagi” (Translation: “Just come close baby, show me your face/I began to get drunk without drinking, friends”) (Zee Music Company, 2017, 00:01:55–00:02:04). Here, the woman's face is metaphorically compared to an intoxicant, suggesting that male desire is both involuntary and overwhelming when confronted with female beauty. This metaphor frames the woman not as a subject but as an object with the power to affect male emotions and behaviors simply through her appearance. Another significant element that highlights the commodification of the woman is the use of the word “brand” in the lyrics (Zee Music Company, 2017, 00:01:39–00:01:42). It implies that her identity and desirability are tied to notions of market value and consumer culture. She is positioned as a commodity to be consumed, admired and ranked within a competitive landscape of attractiveness. In “Engine Ki Seeti” and “Genda Phool,” we have camera shots explicitly focusing and highlighting body parts of the woman as they dance as well (Kashyap, 2014; 00:33:47–00:34:07; Badshah, 2020; 00:01:21–00:01:25).

#### 4.2.2 Gendered representation through choreography and costume

Beyond the lyrics and visuals, choreography plays a crucial role as well. Song-and-dance sequences are central to Bollywood cinema (Dwyer, 2014) and the movements, formations, and staging often highlight and reinforce gender dynamics, positioning women as objects of spectacle. In all the four songs, we see a commonality in the dance move, there is a lot of chest thumping and hip swaying. While Bollywood dance is typically energetic and expressive, in these songs, dance is used deliberately to highlight sensuality and physical allure. Chest and hip are the parts which marks womanhood or woman reproductivity. This is used to present lustful representation of womanhood. In “Engine Ki Seeti”, the choreography itself visually communicates male desire. When the female lead begins to move her hips, the male background dancers respond by shaking their heads rapidly in circular motion, suggesting that her movements drive them crazy (Kashyap, 2014, 00:34:01–00:34:08). This choreography, enhanced by suggestive lighting and camera angles, eroticizes her body and positions her as the central object of visual and lyrical attention. The costuming further reinforces this gendered spectacle. Across all the songs, “Engine Ki Seeti,” “Pallo Latke,” “Jugni,” and “Genda Phool,” recurring colors such as white, red, and pink are used to signify femininity. These color choices are deeply symbolic: pink, commonly associated with softness and nurturing, reflects cultural notions of idealized womanhood (Ma and Wang, 2024); red, traditionally linked with sensuality, fertility, and marital status, conveys passion and desirability (Prokop and Hromada, 2013); while white connotes purity and virtue (Cherry, 2025), echoing patriarchal ideals of the “saintly” woman. For instance, the female protagonist in “Engine Ki Seeti” wears a costume dominated by pink and in “Pallo Latke,” the woman's veil is pink as well. Similarly, the leads in “Jugni” and “Genda Phool” wear white and red sarees, embodying a blend of seduction and sanctity. Through these recurring visual motifs, Bollywood song sequences construct and reinforce gendered meanings. The repeated use of red, pink, and white does not merely serve aesthetic purposes, it visually encodes social attitudes toward femininity and shapes how audiences perceive women on screen.

#### 4.2.3 Dominant male voice

Except in “Engine Ki Seeti,” all the other three songs examined in this study typically has a male voice that describes, praises or comments on the woman. This narrative structure reinforces the idea that women are often positioned as subjects to be looked at and interpreted through the lens of male desire. Throughout the song “Jugni,” it is a male singer who provides the narrative voice, singing about the woman rather than allowing her to express herself. This one-sided narration underscores a broader pattern in many Bollywood song sequences, where women are visually displayed but verbally silenced. At one point, in “Pallo Latke,” the male singer identifying himself as famous, instructs the woman to “behave properly” (Zee Music Company, 2017, 00:01:42–00:01:46). This lyric reveals how male characters often impose their own standards of appropriate female conduct, reflecting wider societal norms in which men feel entitled to regulate women's actions, especially in romantic or public spaces.

#### 4.2.4 Masculine desire and spectatorship

The behavior and gaze of male characters in these music videos further intensify the gendered power dynamic on screen. In “Jugni”, the opening scene immediately establishes how the female body will be framed throughout. The woman appears in silhouette, motionless like a statue, as several male hands reach toward her (T-Series, 2011; 00:00:00–00:00:05). This imagery vividly conveys collective male desire and objectification, stripping the woman of agency and presenting her as an emblem of male fantasy. The accompanying lyrics, “Munde labh de phirde chance” (Translation: “The boys are hunting for a chance with her”), reinforce this idea by depicting men as active pursuers and women as passive objects of pursuit (T-Series, 2011, 00:00:00–00:00:06). The term “hunting” invokes a predatory tone, underscoring the dominance of the male gaze. A similar visual dynamic appears in “Pallo Latke,” where the male lead winks at the woman multiple times (Zee Music Company, 2017, 00:02:08–00:03:22). The act of winking, often framed as playful flirtation, functions here as a display of masculine confidence and control, while the woman's blushing response reinforces her position as the object of his gaze. Through the lyrics, she expresses discomfort at his persistent and suggestive looks (Zee Music Company, 2017, 00:02:06–00:02:45), revealing her vulnerability and lack of agency. This tension is heightened using close-ups and slow-motion shots particularly one where the man lowers his sunglasses and winks directly at the camera. By the end of the song, the camera lingers in a slow upward tilt from the woman's chest to her face, followed by a close-up of the man winking, which is a clear instance of sexual innuendo that symbolically calls for intimacy (Zee Music Company, 2017, 00:03:16–00:03:21). Despite the underlying implications of physical harassment in society, the sequence is stylized and romanticized, transforming a problematic gesture into one perceived as charming or desirable. Such portrayals blur the boundaries between teasing and consent, perpetuating cultural narratives that normalize the male gaze and trivialize women's discomfort (Mathew, 2023). Consequently, these representations not only reflect but also reinforce societal attitudes that conflate harassment with romance and male dominance with desirability.

In “Genda Phool,” Badshah's appearance in non-ethnic attire situates him as the embodiment of the modern male gaze within a traditionally coded aesthetic space. The juxtaposition of his urban image against a folk-inspired backdrop creates a tension between modernity and tradition. Jacqueline Fernandez is introduced through slow, sensual movements, her face revealed with deliberate pacing as Badshah gazes at her in awe, removing his sunglasses to signify desire and admiration. This framing positions the female performer as an object of visual pleasure, aligning with the conventions of the male gaze (Mulvey, 1975). The song further amplifies eroticism through symbolic props, gestures, and wordplay. Badshah wields a cricket bat engraved with the words “bad boy” and sings, “Khelta nahi cricket wricket, par le loon teri wicket wicket” (Translation:“I don't play cricket, but I'll take your wicket”; Badshah, 2020, 00:00:36–00:00:44). The use of a sports metaphor functions as a sexual innuendo, equating conquest in the game with seduction and possession in romance. Such playful metaphors naturalize male dominance and reinforce the trope of flirtatious pursuit. Similarly, when Badshah pretends to play Jacqueline like a guitar, the choreography itself turns her body into a performative prop, symbolizing total objectification (Badshah, 2020, 00:01:05–00:01:08). Also, his gestures such as licking his lips and miming the act of consuming her while singing “tastes like sugar” (Badshah, 2020; 00:01:34–00:01:36) visually translate desire into a form of predation, collapsing sensuality into consumption. Likewise, in “Engine Ki Seeti,” the male character's act of watching the woman dance from a distance encapsulates the dynamics of spectatorship (Kashyap, 2014, 00:35:15–00:35:38). His hidden observation places him and, by extension, the audience in the position of a voyeur. Even though the male voice is absent in the lyrics, the camera's alignment with his gaze ensures that the male perspective dominates the visual narrative. This interplay of silence, surveillance, and framing transforms the woman's dance into a site of erotic display.

Ironically, except for “Engine Ki Seeti,” other songs analyzed feature women in significant creative roles as choreographers and directors. This reveals the complex entanglement between authorship and representation in Bollywood. Despite women's increasing visibility behind the camera, the visual and narrative frameworks continue to reproduce patriarchal aesthetics. Female creators, consciously or otherwise, often operate within an industry that normalizes the objectification of women's bodies. As a result, the internalization of the male gaze becomes not only a cinematic convention but also a creative limitation, where women participate in reproducing the very gaze that confines them.

### 4.3 White skin obsession and westernization

Bollywood has long been obsessed with fair skin as the dominant marker of beauty (Ahmed, 2022). This obsession is clearly reflected in these adapted folk songs, where the women portrayed are almost invariably fair-skinned. The repeated use of fair-skinned actresses reinforces a narrow and exclusionary beauty ideal, positioning light skin as synonymous with desirability, modernity, and success. These portrayals are not isolated; rather, they continue a long-standing tradition within Indian cinema where beauty and virtue are visually coded through fairness. In “Pallo Latke,” the lyrics themselves participate in constructing and legitimizing this ideal. The singer compares the woman's facial beauty to that of popular Bollywood heroines such as Kareena Kapoor and Katrina Kaif (Zee Music Company, 2017, 00:01:46–00:01:55), both known for their fair complexion and conventional attractiveness. This lyrical comparison situates the female subject within a hierarchy of beauty defined by celebrity culture and mediated through Bollywood's visual grammar. By invoking these names, the song reinforces the idea that a woman's appeal is measured against established film industry icons who embody these standardized notions of beauty. Furthermore, such representations reflect a deeper social conditioning where fair skin continues to be equated with purity, sophistication and higher social status (Dixit, 2019). The repetition of fair-skinned female figures in music videos and lyrics subtly teaches audiences that beauty is not diverse but uniform, excluding darker-skinned women from mainstream representations of desirability.

The increasing use of English words in Bollywood lyrics signifies the changing social and cultural landscape of India. It reflects globalization, urbanization and the growing influence of Western culture on Indian society (Shakoor et al., 2015). The blending of Hindi with English, often called “Hinglish,” has become a way for Bollywood to connect with modern, urban audiences who are comfortable switching between languages in everyday life. This linguistic mix also represents aspiration and modern identity. Using English words in songs often signals cosmopolitanism and youthfulness. They appeal to the middle and upper classes who associate English with education, modernity, and progress. At the same time, this practice can also be seen as a form of cultural hybridity, showing how Indian popular culture merges traditional and global influences. However, this trend also has a critical side. It highlights how English has become a marker of status in India. The frequent use of English words may alienate rural or non-English-speaking audiences and reinforce class divisions. Moreover, it sometimes dilutes the poetic richness of Hindi and regional languages, replacing cultural depth with commercial appeal. In the case of folk song adaptations, the incorporation of English words contributes to the loss of their original essence (Table 1). What were once deeply rooted cultural expressions of community and identity are now repackaged for a globalized and upper–middle–class audience. The linguistic shift indicates that the target listeners are no longer native or rural audiences who identify with the folk tradition, but rather urban, English-speaking consumers whose tastes are shaped by Westernized ideals. This transformation underscores how Bollywood's adaptation culture prioritizes global marketability over the preservation of cultural heritage. The following table shows all the English words (except proper nouns, articles, and prepositions) used in the lyrics of these adapted folk songs in Bollywood.

**Table 1:** Incorporation of English vocabulary in the lyrics of all Bollywood adapted folk songs.

Song	English words in the Lyrics
“Jugni”	Naughty, Western, Dance, Gentleman, Romance, Chance, Degree, College, Fashion, Tension, Life, Favorite, Motor Bike, Heavy Vehicle, Bus, Over smart, Ticket, Foreign, and Final
“Engine Ki Seeti”	Engine, Bum, and Driver
“Palo Latke”	Piece, Face, Brand, Down, Beauty, Close, Baby, Non-Stop, Craze, and Chocolate
“Genda Phool”	Bum, Butterfly, Body, Butter, Come, Kick, Ticket, Cricket, Wicket, Wind, Yeah, Baddest m^*^therf, Heartbeat, Missing, Look, Me, Eye, Love, Take, Sky, Promise, Water, Never, Down, Your, Gonna, Lie, and Boy

#### 4.3.1 Hybridity and the construction of the modern Indian woman

The use of English words in the adapted folk songs does more than reflecting linguistic change, it represents a deeper cultural hybridity within Bollywood's portrayal of gender and modernity. This hybridity extends to how women are imagined and represented on screen. The insertion of English terms and westernized imagery transforms the traditional Indian woman into a hybrid figure, one who embodies both modern aspirations and cultural rootedness. In “Pallo Latke,” this tension is explicitly conveyed through the juxtaposition in the lyrics: “face firangi, ghoonghat desi” (Zee Music Company, 2017, 00:01:37–00:01:40). The phrase signifies the dual expectations placed upon Indian women in a postmodern society, they are encouraged to appear modern, cosmopolitan and confident (“face firangi”) while remaining modest and culturally grounded (“ghoonghat desi”). This duality encapsulates the paradox of contemporary femininity in India, where modernization and tradition coexist uneasily within the same body (Pranati, 2025). The woman is expected to be independent yet restrained or westernized yet “respectably Indian.”

Similarly, in “Jugni,” the female protagonist is described in terms that merge traditional admiration with Western attributes. The lyrics emphasize that she is well-educated, rides a motorcycle, dances to western music and speaks fluent English (T-Series, 2011, 00:01:28–00:01:59), all characteristics that signify the modern Indian woman's social evolution. Yet, these qualities are filtered through the male gaze as the man's narration defines what makes her desirable. The “modern” woman, therefore, remains an object constructed for male consumption, not a subject of her own identity. Her confidence and global sensibility are celebrated, only, as they fit within patriarchal frameworks of attractiveness. This process of constructing modern Indian femininity is further reinforced visually. In “Genda Phool,” for example, Jacqueline Fernandez's styling fuses Indian and Western aesthetics. She wears a high-slit saree, a traditional garment altered to enhance sensuality, while background dancers appear in red cabaret costumes (Badshah, 2020, 00:01:21–00:02:30). This visual hybridity symbolizes the westernization of Indian femininity, where traditional clothing and gestures are transformed to fit the aesthetics of global pop culture. The saree, once associated with modesty, is reimagined as an emblem of eroticism, reflecting how Bollywood commercializes cultural symbols to appeal to globalized audiences.

Ultimately, the portrayal of the “modern Indian woman” in these songs embodies a form of postcolonial mimicry (Bhabha, 1994). The fusion of Hindi and English (Hinglish), western fashion and Indian motifs creates a woman who is neither entirely traditional nor fully global. However, rather than liberating her, this hybrid identity often serves male-centered visual pleasure and consumer culture. The modern woman becomes a symbol of India's global modernity, yet she remains bound by patriarchal and capitalist frameworks that dictate how she should look, behave and desire.

## 5 Conclusion

This study has examined how Bollywood adaptations of folk songs function as sites of gendered transformation through narrative shifts, visual spectacle and cultural hybridity. By analyzing four widely circulated adaptations, the paper demonstrates that these songs do not merely preserve folk traditions but actively reinterpret them within commercial and patriarchal frameworks. The findings reveal three key patterns. First, community-centered narratives embedded in folk songs are reoriented toward individualized romance and spectacle. Second, female objectification persists across adaptations through camera techniques, choreography and lyrical framing aligned with the male gaze. Third, cultural hybridity, expressed through Hinglish lyrics, westernized aesthetics and colorist casting, transforms folk authenticity into a commodified cultural product. While these adaptations occasionally present moments of female agency, such representations are often subsumed within visual regimes that prioritize consumption and male pleasure. Thus, Bollywood's engagement with folk traditions emerges as both preservative and distortive, circulating cultural forms while simultaneously reshaping their meanings. By integrating feminist film theory with adaptation studies, this paper highlights the need to critically examine popular cultural forms as ideological practices that mediate gender, identity, and cultural memory in contemporary India.

## 6 Implications

This paper calls for action among academics, filmmakers and society at large by demonstrating that Bollywood's adaptation of folk songs is not simply a creative or commercial exercise but an ideological practice that reshapes cultural meaning. The analysis reveals how folk songs, originally rooted in communal memory, women's lived experiences and regional identity, undergo significant narrative and visual transformation when adapted for Bollywood. While these adaptations occasionally appear to grant women agency through bold choreography, confident performances, or hybrid aesthetics, they simultaneously reproduce subtle and overt forms of female objectification that often go unnoticed by general audiences. Such contradictions underscore the need for greater critical awareness, especially as Bollywood continues to adapt folk materials. A recent example is the commercially popular folk adaptation “Panwadi” in *Sunny Sanskari Ki Tulsi Kumari* (2025), which again showcases how traditional motifs are glamorized for mass appeal. Existing scholarship has richly explored objectification in item songs, yet little attention has been paid to how similar patriarchal framings infiltrate folk adaptations, songs not originally intended to be erotic or consumerist. By foregrounding this gap, the paper contributes to both gender studies and adaptation studies, showing how the processes of recontextualization, hybridization and commercialization intersect with the male gaze. It also urges filmmakers to reconsider how they credit, represent and ethically adapt folk traditions and invites educators to use such analyses to build media literacy around cultural appropriation, gendered representation and the politics of popular music.

## 7 Limitations

Despite its contributions, the study has certain limitations. Bollywood contains numerous folk adaptations, such as “Chaiyya Chaiyya” (1998), “Nimbooda Nimbooda” (1999), and more recent songs like “Morni Banke” (2018), but this paper analyzes only four 21st century adaptations. This selective focus supports depth but limits the breadth and generalizability of the findings. Furthermore, the study centers exclusively on textual and visual analysis, lyrics, choreography, cinematography and costume, without drawing upon audience reception data or empirical evidence such as surveys, social media discourse or interviews with viewers, filmmakers, or folk artists. As a result, while the paper maps how gendered meaning is constructed on-screen, it does not measure how audiences interpret these meanings or how communities perceive the adaptation of their cultural materials. Nonetheless, these limitations identify productive directions for further scholarship.

## 8 Future scope

This study serves as a foundation for expanded research on folk song adaptations within Bollywood and other Indian film industries. Future research can examine a larger corpus of adapted folk songs across decades and regions to determine whether the patterns identified are consistent across regional cinemas. Empirical audience reception studies involving interviews, focus groups or digital ethnography would help reveal how different communities interpret these adaptations and whether objectifying portrayals influence attitudes toward gender, tradition or modernity. Production-side ethnographic research like speaking with composers, choreographers, folk performers and label executives, could illuminate the economic and institutional motivations behind adaptation choices, as well as the recurring issue of uncredited folk creators. Comparative and intersectional analyses exploring caste, class, religion, or regional identity within folk adaptations would further deepen understanding of how marginalized voices are represented or erased. Finally, practice-based and policy-oriented research can explore ethical adaptation models, including co-authorship with community artists, transparent crediting processes and cultural impact assessments. Such expanded work would not only advance academic debates but also offer practical guidelines for more responsible and culturally sensitive cinematic adaptation.

## Data Availability

The original contributions presented in the study are included in the article/supplementary material, further inquiries can be directed to the corresponding author.
